# Rectal Surgery

**Published:** 1898-02-05

**Authors:** 


### RECTAL SURGERY.

At the last meeting of the Medical Society, Mr. Thomas Bryant read a paper on rectal surgery, and, among other things, insisted on the importance of making a proper examination in all cases in which complaint was made of pain or discomfort at or about the anus. He expressed surprise at the frequency with which fissure or painful ulcer of the rectum was overlooked for want of such an examination. Mr. Alfred Cooper illustrated this point by relating the case of a gentleman who had previously consulted no fewer than eight medical men, six of whom had not examined him at all, while the two others said he was not suffering from fissure. As a matter of fact there was a bone impacted in the rectum, which he removed. The necessity for routine examination was also illustrated by Mr. Battle, who related the case of a woman, whom he had previously seen with a syphilitic stricture of the rectum, who returned later with partial obstruction. Not unnaturally he expected to find that the stricture had closed, but as a matter of fact he found three sovereigns impacted there, which he withdrew. Mr. Goodsall pointed out that 90 per cent. of diseases of the rectum were situated within two inches of the anus, and that many men made the mistake of introducing the finger too far to begin with. The President, Mr. Pearce Gould, gave a useful "tip" for finding the starting points of the various ramifications of a fistula. He said that one advantage of scraping the track of a fistula was that the granulation tissue lining the collateral ramifications bulged and so indicated the points where they branched off.

